# Role of Interleukin-38 in Chronic Inflammatory Diseases: A Comprehensive Review

**DOI:** 10.3389/fimmu.2018.01462

**Published:** 2018-06-22

**Authors:** Wang-Dong Xu, An-Fang Huang

**Affiliations:** ^1^Department of Evidence-Based Medicine, School of Public Health, Southwest Medical University, Luzhou, China; ^2^Department of Rheumatology and Immunology, The Affiliated Hospital of Southwest Medical University, Luzhou, China

**Keywords:** interleukin-38, inflammation, signaling pathway, immune cell, autoimmunity

## Abstract

Interleukin (IL)-38 is the newest member of the IL-1 family. It can bind to several receptors, regulate the generation, function of inflammatory cytokines through the downstream signaling pathways. IL-38 is expressed in several tissues, such as placenta, heart, and brain. It is involved in a wide variety of diseases, including chronic inflammatory diseases. In this review, we discuss the expression and biological functions of IL-38, especially the role in rheumatic autoimmune diseases. Collection of the information may improve the understanding of IL-38, and may give potential for theoretical basis for clinical trials and drug development in the future.

## Introduction

Interleukin (IL)-38 was originally cloned as an IL-1 family (IL-1F) cytokine, and named IL-1HY2 in 2001 (1). The IL-1F is composed of 11 members (2). IL-1F5, IL-1F6, IL-1F8, IL-1F9, and IL-1F10 recently have been renamed IL-36 receptor antagonist (IL-36Ra), IL-36α, IL-36β, IL-36γ, and IL-38, respectively (3). There are four exons in human IL-38 gene, which is located within the IL-1 gene cluster on human chromosome 2p13 near the IL-1R antagonist (IL-1Ra) and IL-36Ra gene (4). The IL-38 gene is located 49,479 bp upstream from IL-1Ra gene on the same DNA strand (5). The primary translated product is an IL-38 precursor, 152 amino acids in length and with 16.9 kD molecular mass (6). It is expressed in numerous tissues, such as heart, placenta, fetal liver, skin, spleen, thymus, and in proliferating B-cells of the tonsil. As is typical of the IL-1F, IL-38 lacks a signal peptide and caspase-1 consensus cleavage site. However, the natural N terminus for IL-38 is still unclear. Similar to the crystal structure of IL-1Ra and IL-1, IL-38 displays a 12-β-stranded trefoil structure and shares. It has been found that IL-38 shares 41% homology with IL-1Ra, 43% homology with IL-36Ra, lower homology (14–30%) with IL-1β and other IL-1 subfamilies (5, 7). It has a three-dimensional structure similar to IL-1Ra (7). In mammalian Chinese hamster ovary cells, recombinant IL-38 protein was synthesized into two forms, a major form at 25 kD and a minor form at 17 kD. Recent findings suggested that expression of IL-38 was abnormal in chronic inflammatory diseases, especially rheumatic autoimmune diseases, such as systemic lupus erythematosus (SLE) (8, 9), rheumatoid arthritis (RA) (10, 11), psoriasis (10, 12), and inflammatory bowel diseases (IBD) (10). Similarly, gene polymorphisms of IL-38 were reported to correlate with spondyloarthritis, RA, and psoriatic arthritis susceptibility (13–15). In addition, functional analysis confirmed that IL-38 overexpression or knockdown significantly involves in the pathogenesis of these diseases (9, 16, 17). Therefore, the strong association of this inflammatory cytokine with chronic inflammatory diseases on multiple levels promotes us to summarize the recent findings related to crucial nature of IL-38 with respect to these diseases gaining attention for its regulatory capability in these disorders. In this review, we will systematically discuss the biologic role of IL-38 in different cells, and elucidate the mechanisms that IL-38 contributes to the diseases.

## Biologic Functions of IL-38

### IL-38 as an Immune Modulator

**Figure 1:**
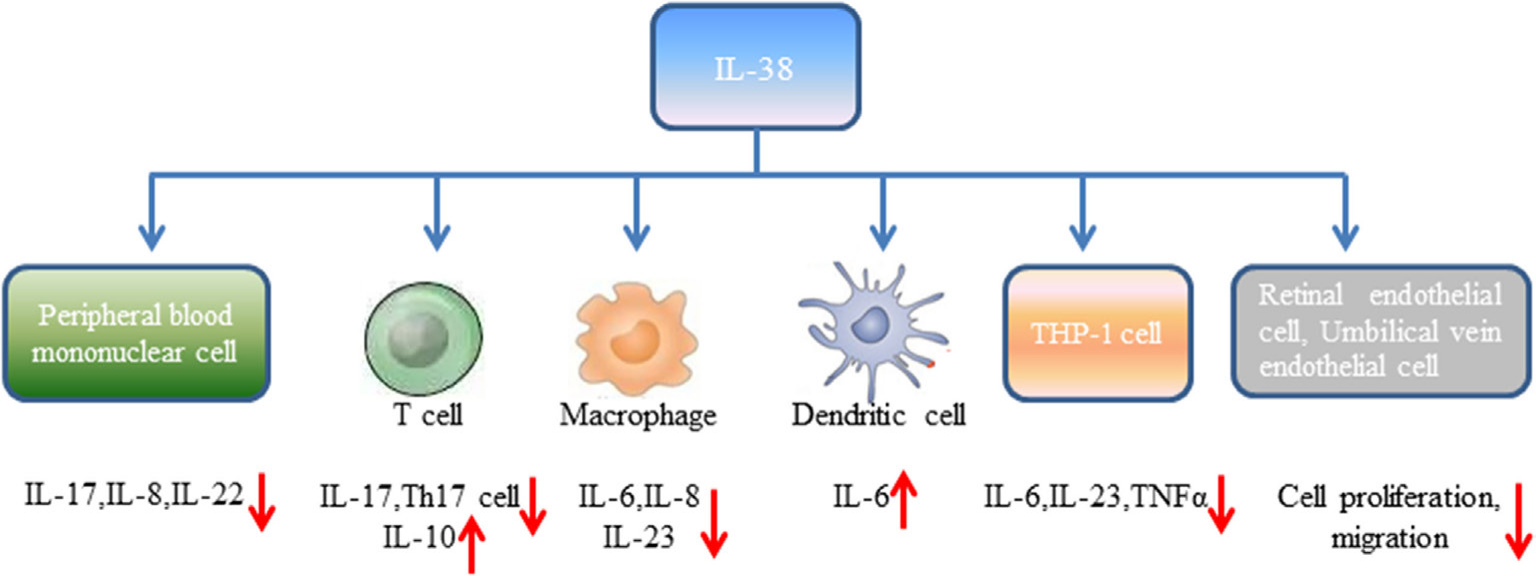
Biological effects of interleukin-38 on distinct cell types.

Human peripheral blood mononuclear cells (PBMCs) from healthy volunteers cultured with heat-killed *Candida albicans* reflected by consistent production of IL-17, IL-22, and IFN-γ (18). However, expression of IL-17, IL-22 induced by *C. albicans* was significantly reduced in the presence of IL-38. It has been widely accepted that controlled disintegration of cells, apoptosis can avoid the inflammatory response and contribute to inflammation resolving. On the contrary, cell death involving cell lysis, such as necrosis and necroptosis, may lead to inflammation through generation of pro-inflammatory molecules (19). Mora et al. found amounts of IL-38 in the supernatant of apoptotic A549 lung or MDA-231 breast cancer cells (apoptotic cell-conditioned media, ACM) compared with that in the necrotic cell-conditioned media (20). T cells cultured with ACM from macrophages reduced IFN-γ, IL-10 secretion by T cells, and slightly increased IL-17 levels, while lack of IL-38 in ACM significantly promoted IL-17 generation, downregulated IL-10 expression (20). By contrast, overexpression of IL-38 in ACM blocked IL-17 production from T cells, indicating that IL-38 from apoptotic cells restricts macrophage-dependent generation of Th17 cells (Figure 1).

Peripheral blood mononuclear cells stimulated with IL-36γ in the presence of IL-38 showed reduced expression of IL-8. Similarly, PBMCs stimulated with IL-36γ under the antagonist IL-36Ra treatment downregulated IL-8 generation, supporting the concept that IL-38 suppresses IL-36γ-induced IL-8 (18). Addition of lipopolysaccharide (LPS) to macrophages promoted IL-6, IL-8 production, however, stimulation of human macrophages with ACM generated from IL-38 overexpressing A549 cells yielded decreased expression of IL-6, IL-8. Macrophages treated with ACM of IL-38 knockdown A549 cells led to elevated IL-6, IL-8 expression (20). Human monocyte-derived dendritic cells with LPS stimulation in the presence of IL-38 observed increased expression of IL-6 production (18). When THP-1 cells transduced with lentiviral particles that overexpressed human IL-38 gene (THP-1-IL-38), there was significantly reduced IL-6 and IL-23p19 expression after LPS stimulation (16). M1 macrophages from healthy volunteers cultured with media from THP-1-IL-38 cells showed reduced expression of IL-6, IL-23 (16). Interestingly, there was detectable IL-38 (17–18 kD) in the supernatants of THP-1-IL-38 cells. Media from THP-1-IL-38 cells strongly downregulated IL-6, TNFα, and IL-23 production when THP-1 cells were stimulated with LPS. Moreover, THP-1 cells treated with media from the epithelial HEK cells that overexpressed IL-38 downregulated IL-6 secretion, suggesting that the anti-inflammatory effect of IL-38 may be autocrine or paracrine (16).

**Figure 2:**
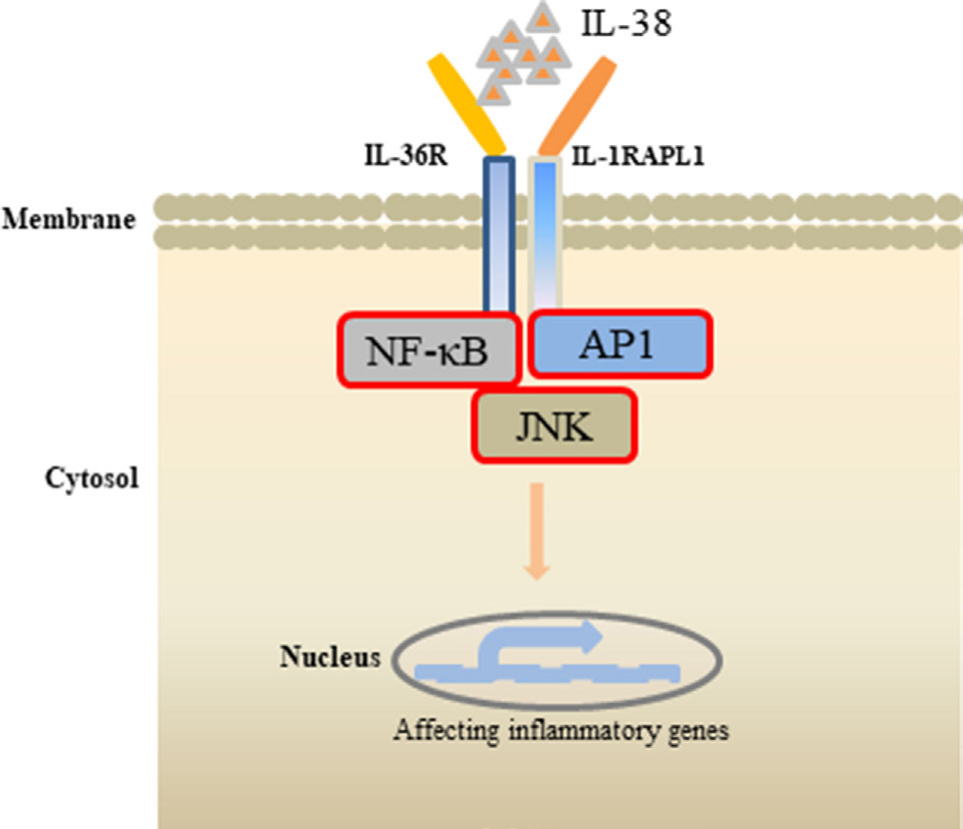
Effects of interleukin (IL)-38 binding. IL-38 binds to the receptors IL-1RAPL1 or IL-36R, exerts anti-inflammatory properties by inhibiting the downstream signaling pathways, such as NF-κB, AP1, JNK, and, therefore, regulates the secretin of inflammatory cytokines.

Receptor study indicated that IL-38 is able to bind to IL-1R type I, IL-1R accessory protein-Fc, IL-18 receptor α chain-Fc, and IL-1Rrp2-Fc (IL-36R) (18). PBMCs cultured with *C. albicans* in the presence of IL-1Ra indicated a strong reduction of IL-17 and IL-22, suggesting that IL-38 binds to IL-1Rrp2, and, therefore, affects the Th17-related cytokines secretion. ACM of IL-38 knockdown A549 cells promoted AP1 activation in macrophages. Interestingly, IL-38 competed with the receptor IL1RAPL1 in macrophages, and bound to IL1RAPL1 (20). Knockdown IL1RAPL1 gene in macrophages significantly reduced IL-6, IL-8 secretion. However, reduced cytokines generation was reversed when macrophages were stimulated with ACM from IL-38 overexpressed cells, indicating that IL-38 may inhibit IL1RAPL1 signaling pathway, and, therefore, leaving no room for knockdown-dependent reduction. IL1RAPL1 overexpressed in HEK 293 T cells activated AP1, increased JNK phosphorylation. IL1RAPL1 overexpression also activated the IL-6 promoter, but was abolished when AP1, NF-κB binding sites were mutated. However, IL1RAPL1 overexpressed cells treated with IL-38 inhibited AP1 activity, reduced JNK phosphorylation (20), demonstrating the suppressive role of IL-38 (Figure 1). Collectively, these data showed that IL-38 binds to the receptors and regulates the inflammatory cytokines generation *via* the downstream signaling pathways (Figure 2).

### IL-38 Performs in Angiogenesis

Angiogenesis is a process of new blood vessels sprouting from pre-existing vasculature, and pathological angiogenic signaling may lead to disordered neovascularization. Neonatal mice administrated with IL-38 showed reduced vascular area, vessel density, and tip cell numbers compared with the control mice (21). In the mice of oxygen-induced retinopathy (OIR), hypoxia-triggered angiogenic response induces the formation of pathological neovessels and neovascularization tufts, whereas IL-38 administration abolished the effects. Supplementation of IL-38 in human retinal endothelial cells (HRECs) and human umbilical vein endothelial cells (HUVECs) significantly decreased vascular endothelial growth factor-induced HREC proliferation in comparison to the controls, and decreased the proliferation of HUVECs (21). Similarly, the healing area of HRECs in IL-38-treated cells was decreased, and addition of IL-38 reduced the migration of HUVECs. These findings revealed that IL-38 is able to attenuate endothelial cell proliferation, migration, and suppress pathological angiogenesis (21) (Figure 1).

## Correlation Between IL-38 and Chronic Inflammatory Diseases

### Rheumatoid Arthritis

Rheumatoid arthritis is a systemic autoimmune disease with severe joint inflammation and destruction associated with an inflammatory environment (22). IL-38 mRNA levels were strongly elevated in the synovial tissue of patients with RA compared to patients with osteoarthritis (OA), and associated with the total leukocyte counts in the synovial fluid, especially the number of monocytes, and expression of IL-1β, IL-1Ra, CCL3, and CCL4 (10, 11) (Table 1).

**Table 1:** Aberrant expression of IL-38 in rheumatic autoimmune diseases.

Animal models/patient data	Diseases	Increase/decrease compared with controls	Reference
Serum levels of IL-38 in patients	SLE	Increase	(9)
IL-38 mRNA expression in spleen, thymus from mice models	SLE	Decrease	(8)
mRNA and protein levels of IL-38 in labial salivary from patients	pSS	Increase	(31)
Serum concentrations of IL-38 in patients	Psoriasis	Increase	(12)
IL-38 mRNA expression in skin from mice models	Psoriasis	Decrease	(10, 35)
IL-38 mRNA levels in synovial fluid from patients	RA	Increase	(10, 11)
IL-38 mRNA expression in joints from mice models	RA	Increase	(10, 11)
mRNA levels of IL-38 in colon from patients	CD	Increase	(10)
mRNA levels of IL-38 in colon from mice models	CD	Increase	(10)

*SLE, systemic lupus erythematosus; pSS, primary Sjögren’s syndrome; RA, rheumatoid arthritis; CD, Crohn’s disease*.

Experimental arthritis (collagen-induced arthritis, CIA) was induced in male DBA/1J mice with bovine type II collagen and Freund’s incomplete adjuvant, which is a prototype model of RA and shares many clinical and histopathological similarities to human RA (23). In joints from CIA mice, there was significantly increased mRNA expression of IL-38 (10). It is known that K/BxN arthritis is an IgG autoantibody mediated immune complex mouse arthritis that leads to significant joint tissues inflammation after injection of arthritogenic serum (24). Takenaka et al. reported significantly increased mRNA expression of IL-38 in mice joints during autoantibody-induced arthritis (11). Boutet et al. subcloned the full length mouse IL-38 cDNA into the SSV9scCMV plasmid, under the control of the cytomegalovirus promoter, to generate a double-stranded adeno-associated virus, serotype 2/8 (AAV-IL-38) (16). IL-38 was persistently expressed in the joints of CIA mice after intra-articular injection of AAV-IL-38, and was mainly located in injected ankles, at lower levels in inguinal lymph nodes, spleen, and liver (16). CIA mice injected with AAV-IL-38 showed significantly reduced expression of Th17 cytokines (IL-17, IL-23p19, IL-22), chemokine (C-X-C motif) ligand 1 (CXCL1), and receptor activator of nuclear factor kappa-B ligand (16). IL-38 overexpression also downregulated IL-10, TNFα levels. When IL-38-deficient (IL-38^−/−^) mice induced K/BxN serum transfer arthritis, IL-38^−/−^ mice showed significantly elevated expression of IL-1β, IL-6 in the ankle joints as compared to control mice (11). CIA mice treated with AAV-IL-38 significantly reduced the clinical score, arthritis incidence. In K/BxN arthritis mice, administration of AAV-IL-38 also showed an anti-inflammatory effect. Interestingly, AAV-IL-38 injection dramatically reduced the histological inflammatory score during the resolution phase of inflammation. Within the inflammatory synovial area, the density of Iba1^+^ monocytes/macrophages was strongly decreased after AAV-IL-38 treatment (16). Similarly, IL-38^−/−^ K/BxN mice exhibited significant exacerbation of the clinical scores during arthritis, and histomorphometric quantification of the arthritic changes in the joints found significant increases in inflammation and bone erosion score (11). The findings above indicated that expression of IL-38 was increased in arthritis patients and mice models, and IL-38 plays a negative role in the pathogenesis of arthritis.

### Systemic Lupus Erythematosus

Systemic lupus erythematosus is a highly heterogeneous disorder, characterized by differences in autoantibody profile, serum cytokines, and a multi-system involvement commonly affecting the skin, renal, musculoskeletal, and hematopoietic systems clinical manifestations involving (25, 26). Regarding the observations in patients, IL-38 protein concentrations were significantly higher in SLE patients at baseline, at the first and second visit when compared to that in healthy controls in a follow up study (9) (Table 1). SLE patients with nephritis, central nervous system involvement showed higher protein expression of IL-38 compared with that in SLE patients without the clinical characteristics (9). Interestingly, patients with active disease showed higher serum IL-38 concentrations compared with that in patients with inactive disease, healthy controls. It is notable that IL-38 protein expression at baseline or time-adjusted IL-38 concentrations was dramatically higher in patients who fulfilled criteria for persistently active disease in subsequent visits compared with those who did not, suggesting that serum IL-38 levels were predictive of subsequent disease activity (9). Elevated serum levels of IL-38 in SLE patients were decreased after treatment (27). IL-38 silencing significantly promoted the generation of IL-6, chemokine CCL2, and APRIL in unstimulated SLE PBMCs and by cells activated with agonists of toll-like receptor (TLR)-7 (imiquimod) as well as TLR-9 (CpG ODN) (9).

MRL/lpr mouse model is a spontaneous lupus model. The mice have a loss-of-function lymphoproliferation (lpr) mutation within the gene encoding Fas, a cell-surface protein that mediates apoptosis, recapitulate many features of human lupus (28). They are characterized by lymphoproliferation, enlarged lymph nodes (lymphadenopathy), and some of the mice develop arthritis. Serologically, the mice display hyperimmunoglobulinemia, high antinuclear antibodies, high anti-double-stranded DNA antibodies, and anti-small nuclear ribonucleoprotein antibodies (29). In MRL/lpr mice, mRNA expression of IL-38 in spleen, thymus was lower compared to that in the control mice (8) (Table 1). Injection of IL-38 in MRL/lpr mice significantly downregulated the levels of epigenetically regulated gene expression of Th17 (*Il17a, Il17re, Il21, Rora, Rorc*), whereas treatment with IL-38 strongly increased the expression of regulatory T cells regulated genes *(Foxp3, Ikzf2, Irf4, Irf8*) as compared with the control mice (8). Serum concentrations of IL-6, IL-17, IL-22, CXCL10, IL-1β, IFN-γ, TNFα were significantly decreased in IL-38-treated mice, and expression IL-10 was elevated. In addition, MRL/lpr mice treated with IL-38 showed remission of vessel infiltrate, a marked reduction in skin lesions severity, decreased proteinuria score, glomerulonephritis scores when compared to the control mice dominantly characterized by amelioration of mesangial thickening and proliferation (8). IL-38-treated mice had significantly reduced proportion of splenic Th17 cells, smaller spleen weight, and exhibited a significant decrease in the proportion of CD3^+^CD4^−^CD8^−^ double negative T cells (4). Together, these data suggested that IL-38 attenuated the development of lupus.

### Primary Sjögren’s Syndrome (pSS)

Primary Sjögren’s Syndrome is a systemic autoimmune disease mainly affecting exocrine glands and leading to impaired secretory function. The clinical picture is dominated by signs and symptoms of mucosal dryness and the course of the disease is mild and indolent in the majority of cases (30). In pSS patients, mRNA and protein levels of IL-38 were elevated in minor labial salivary glands compared to that in the non-pSS patients (31) (Table 1). Increased IL-38 expression in the salivary glands of pSS patients was expressed mainly among acinar epithelial cells and infiltrating mononuclear cells (31).

### Inflammatory Bowel Disease

Crohn’s disease (CD) and ulcerative colitis, collectively known as IBD, are chronic inflammatory disorders of the gastrointestinal tract. The group of diseases is due to the interaction of genetic and environmental factors that trigger an unbalanced immune response ultimately resulting in the peculiar inflammatory reaction (32). IL-38 mRNA levels were higher in inflamed colonic biopsies of patients with CD compared to unaffected biopsies from the same patients, and correlated with IL-1β, IL-17A, IL-6 expression (10) (Table 1). Dextran sulfate sodium (DSS)-induced colitis is a well-established model to study human IBD. Colitis was induced in normal C57BL/6J male mice received DSS in the drinking water until loose stools, diarrhea, and macroscopically visible blood appeared (33). In colon isolated from DSS-induced colitis mice, there was higher mRNA expression of IL-38 compared to the control mice (10), suggesting that IL-38 expression was elevated in colitis patients and mice models.

### Psoriasis

Psoriasis is a long-lasting autoimmune disease characterized by patches of abnormal skin (34). Patients with psoriasis had higher serum levels of IL-38 than that in healthy controls (12) (Table 1). Imiquimod (IMQ), a TLR-7 agonist that activates the innate immune response, can induce psoriasis in mice models, and application of the IMQ-containing Aldara cream on mouse skin causes cutaneous inflammation with leukocyte influx and epidermal hyperplasia, resembling human psoriatic lesions (35, 36). Palomo et al. induced psoriasis-like skin inflammation in female IL-38^−/−^ mice and their respective wild-type littermates by application of Aldara cream, containing IMQ on ears (35). IL-38 mRNA was detected in the epidermis and in primary mice keratinocytes, and skin from IMQ-induced inflammation in mice showed reduced mRNA expression of IL-38 compared to control mice (10, 35). When IL-38^−/−^ mice induced psoriasis-like skin inflammation by IMQ, the severity of IMQ-induced skin inflammation, as assessed by recording ear thickness and histological changes, was similar in IL-38^−/−^ and control mice (35). IL-38 deficiency had no impact on IMQ-induced expression of pro-inflammatory mediators in the skin. In addition, after cessation of topical IMQ application, the resolution of skin inflammation was not altered in IL-38^−/−^ mice (35). Therefore, the expression or role of IL-38 in psoriasis still remains to be demonstrated in the future.

### Chronic Obstructive Pulmonary Disease (COPD)

Chronic obstructive pulmonary disease is a complex disease with many patients suffering from cardiovascular comorbidity. Patients with acute exacerbation of COPD showed higher serum levels of IL-38 than that in stable COPD patients (37). COPD patients in acute exacerbation and stable disease activity both showed elevated serum levels of IL-38 than that in healthy controls. Serum levels of IL-38 positively correlated with body mass index, and negatively correlated with C reactive protein (CRP), fibrinogen levels, and the number of acute exacerbations in the past 1 year. Interestingly, the CRP level and the number of acute exacerbations in the past 1 year were factors that can affect the serum levels of IL-38 in patients with COPD (37).

### Oxygen-Induced Retinopathy

Mice exposed to hyperoxia (75% oxygen) induced OIR. Mice with OIR injected with IL-38 significantly suppressed retinal angiogenesis, and attenuated the proliferation, scratch wound healing, and tube formation of vascular endothelial cells induced by vascular endothelial growth factor, suggesting that IL-38 is an antiangiogenic cytokine in angiogenesis-related diseases (21).

## Conclusion

Seventeen years after its discovery, the role of IL-38 is still poorly understood. IL-38 belongs to the IL-1F, and most of the subfamilies are recognized as pro-inflammatory cytokines that can regulate the expression of genes related to inflammatory diseases. However, recent findings showed that IL-38 exerts anti-inflammatory properties, especially on macrophages, by suppressing generation of pro-inflammatory cytokines, leading to reduced Th17 maturation, therefore, making this cytokine of interest for targeting numerous chronic inflammatory diseases, especially rheumatic autoimmune diseases.

Although we are in the beginning of understanding the biology of IL-38 and its role in these disorders, there are several questions still needed to be elucidated. First, the dominant concern about IL-38 is lacking knowledge of its maturation. Studies have revealed that IL-38 needs to be matured so as to gain its biological activity, but the proteases implicated in this process have not been clearly identified (17). Second, IL-38 is able to resolve inflammation, possibly through immune cells such as macrophages, but the identification of its receptors needs further discussion. Third, available evidence has shown the anti-inflammatory effects of IL-38 in arthritis. However, the mechanism of IL-38 performed in preventing bone erosions in arthritis, or the effect on osteoclast and osteoblast differentiation, activity is unknown. Moreover, IL-38 is recognized to inhibit pathological angiogenesis. Pathogenic angiogenesis is related to the development of arthritis. Therefore, whether IL-38 plays an important role in RA angiogenesis needs to be discussed. In addition, most of the current studies found higher expression of IL-38 in rheumatic autoimmune diseases, but how the high expression of IL-38 negatively regulates the pathogenesis of the diseases is not fully understood. Is there a negative feedback mechanism for IL-38?

All together, with knowledge of the mechanisms that IL-38 regulates chronic inflammatory conditions increasing, there may be potential strategies for the development of anti-inflammatory treatments for these diseases and establish a theoretical basis for clinical trials and drug development in the future.

## Author Contributions

W-DX and A-FH wrote the manuscript. All the authors provided overall guidance and reviewed the manuscript.

## Conflict of Interest Statement

The authors declare that the research was conducted in the absence of any commercial or financial relationships that could be construed as a potential conflict of interest.
